# In vitro and in vivo evaluation of thapsigargin as an antiviral agent against transmissible gastroenteritis virus

**DOI:** 10.1186/s13567-024-01359-x

**Published:** 2024-08-02

**Authors:** Yang Li, Yuanyuan Liu, Yunhang Zhang, Chen Tan, Yifei Cai, Yue Zhang, Jianing Chen, Yuguang Fu, Guangliang Liu

**Affiliations:** 1 State Key Laboratory for Animal Disease Control and Prevention, Lanzhou Veterinary Research Institute, Chinese Academy of Agricultural Science, Lanzhou, China; 2 Hainan Key Laboratory of Tropical Animal Breeding and Infectious Disease Research, Institute of Animal Husbandry and Veterinary Medicine, Hainan Academy of Agricultural Sciences, Haikou, China; 3 College of Veterinary Medicine, Xinjiang Agricultural University, Urumqi, China; 4 Molecular and Cellular Epigenetics (GIGA), University of Liege, Liege, Belgium; 5 Human Nutrition and Health Group, VLAG, Wageningen University and Research, Wageningen, The Netherlands

**Keywords:** thapsigargin, intestinal organoids, ERS, antiviral drug, TGEV

## Abstract

Swine enteric coronaviruses (SeCoVs) pose a significant threat to the global pig industry, but no effective drugs are available for treatment. Previous research has demonstrated that thapsigargin (TG), an ER stress inducer, has broad-spectrum antiviral effects on human coronaviruses. In this study, we investigated the impact of TG on transmissible gastroenteritis virus (TGEV) infection using cell lines, porcine intestinal organoid models, and piglets. The results showed that TG effectively inhibited TGEV replication both in vitro and ex vivo. Furthermore, animal experiments demonstrated that oral administration of TG inhibited TGEV infection in neonatal piglets and relieved TGEV-associated tissue injury. Transcriptome analyses revealed that TG improved the expression of the ER-associated protein degradation (ERAD) component and influenced the biological processes related to secretion, nutrient responses, and epithelial cell differentiation in the intestinal epithelium. Collectively, these results suggest that TG is a potential novel oral antiviral drug for the clinical treatment of TGEV infection, even for infections caused by other SeCoVs.

**Supplementary Information:**

The online version contains supplementary material available at 10.1186/s13567-024-01359-x.

## Introduction

The spread of swine enteric coronaviruses (SeCoVs), including transmissible gastroenteritis virus (TGEV), porcine epidemic diarrhea virus (PEDV), porcine deltacoronavirus (PDCoV), and the newly emerged swine acute diarrhea syndrome coronavirus (SADS-CoV), has caused great financial losses in the global pig industry [1, 2]. SeCoVs infect pigs of all ages and cause severe symptoms such as dehydration, vomiting, and acute diarrhea, often resulting in high mortality rates in neonatal piglets [3, 4]. In 2021, PDCoV infection was identified in three Haitian children, highlighting the potential risk of cross-species transmission [5–7].

Immunoprophylaxis is the main strategy for controlling SeCoVs. While vaccinations have significantly restricted the spread of SeCoVs, their effectiveness is continually limited by the emergence of mutant strains. The use of antiviral drugs is a crucial strategy for enhancing the survival rate of hosts during viral infections. The COVID-19 pandemic, caused by severe acute respiratory syndrome coronavirus 2 (SARS-CoV-2), has driven the exploration of novel antivirals. One such compound, thapsigargin (TG, molecular weight of 650.75), which is derived from plants, has demonstrated broad-spectrum antiviral effects against three human coronaviruses in various in vitro and ex vivo cell models [8]. Additionally, TG has been shown to inhibit respiratory syncytial virus and influenza A virus (H1N1) in both immortalized and primary cells [9]. Furthermore, TG has been shown to inhibit single-variant infection and coinfection with different variants of SARS-CoV-2 [10]. These findings indicated that TG is a potential broad-spectrum antiviral against coronavirus infections. However, further preclinical studies are necessary to advance the clinical application of TG as a new antiviral agent.

TG is commonly used in biological research as an inhibitor of the sarcoplasmic and endoplasmic reticulum Ca^2+^ ATPase pump, which effectively induces endoplasmic reticulum stress (ERS) and the unfolded protein response (UPR) [11, 12]. At low cytotoxic concentrations, TG has demonstrated significant efficacy in inhibiting the replication of CoVs, suggesting its potential as a novel anti-CoV drug. Oral administration of TG may be an effective antiviral solution for enteric CoV infections. However, TG has been shown to inhibit proliferation and induce apoptosis in certain cells, including cancer cells [13].

The emerging intestinal organoid model provides a physiological platform for studying organ development, diseases, pathogen invasion, and drug use. Previous research has utilized 3D and 2D porcine intestinal organoids to investigate the interactions between the intestinal epithelium and enteric CoVs [14, 15]. This model offers a valuable approach to assess the potential of TG as an oral antiviral against SeCoVs and its side effects on the intestinal epithelial barrier.

In this study, we demonstrated that TG exhibited significant anti-TGEV effects on IPEC-J2 cells and an intestinal epithelial monolayer culture model derived from porcine intestinal organoids. RNA-seq was performed to determine the underlying mechanism by which TG restrains TGEV replication on organoid monolayers. Further investigation suggested that the oral administration of TG also had anti-TGEV effects on piglets. Taken together, our data revealed that TG is a potential drug candidate for anti-SeCoV treatment.

## Materials and methods

### Cell lines, viruses, and reagents

The TGEV Miller strain was maintained in our laboratory and titrated in ST cells. The PEDV LJX01/2014 strain was isolated and propagated in our laboratory and titrated in Vero E6 cells. ST cells, Vero E6 cells, and IPEC-J2 porcine intestinal epithelial cells were preserved in our laboratory. IPEC-J2 cells, ST cells, and Vero E6 cells were cultured at 37 °C and 5% CO_2_ in high-glucose Dulbecco’s modified Eagle’s medium (DMEM) (Merck, Darmstadt, Germany) supplemented with 10% fetal bovine serum. To evaluate the antiviral activities of TG, ST cells were infected with TGEV at an MOI of 0.1, while IPEC-J2 cells were infected at an MOI of 1. Vero E6 cells were infected with PEDV at an MOI of 0.1. Cells were treated with TG (MedChemExpress, Shanghai, China) at the indicated concentration, while the control groups were exposed to the same volume of DMSO. For EC_50_ assessments, viral RNA copies of virus-infected cells were tested, and the DMSO group was used for normalization. The EC_50_ values were calculated by nonlinear regression analysis using GraphPad Prism 9.4.1.

### Animal experiments

Piglets were purchased from a pig farm and housed in isolated animal rooms. All experimental procedures and animal care protocols were approved by the Guidelines for the Care and Use of Laboratory Animals of Lanzhou Veterinary Research Institute (LVRI), Chinese Academy of Agricultural Sciences, China. Nine piglets were randomly divided into three groups: the control group, the TGEV infection group, and the TG treatment group. To investigate the anti-TGEV effect of TG in neonatal piglets, the piglets in the TGEV infection group and TG treatment group were orally infected with the TGEV Miller strain (3 × 10^7^ TCID_50_ for each piglet). Then, for the TG treatment group, the piglets were orally administered TG (2 μg/kg) at 1 and 12 h post-infection (hpi). For the control group, the piglets were orally treated with the same volume of PBS. At 24 hpi, the piglets were euthanized, and the intestinal samples were fixed with formalin for pathological examination. RNA samples of intestinal tissue were prepared for RT-qPCR analysis. The serum levels of CREA, AST, ALT, and ALB were measured by biochemical parameters (Hitachi High-Tech, Shanghai, 3110).

### Preparation of porcine intestinal organoids and generation of 2D porcine intestinal organoid monolayers

Porcine ileum samples were obtained from the Animal Husbandry and Veterinary Institute of the Hainan Academy of Agricultural Sciences. Porcine intestinal organoids were generated and cultured as described in our previous study [16]. In brief, small intestine tissue was washed with cold PBS. After incubation in 5 mM EDTA buffer, crypts of the small intestines were isolated by a surgical blade and cultured in Matrigel Matrix (Corning, Bedford, USA) domes supported with commercial Organoid Growth Medium (OGM, StemCell, Vancouver, Canada). To develop 2D organoid monolayer cultures, 3D organoids were dissociated with TrypLE™ Express. After dissociation, the organoids were vigorously pipetted to disrupt them into single cells. Single cells from 3D organoids were resuspended in OGM and seeded into a Matrigel precoated 24-well cell culture plate. Confluent 2D organoid monolayers formed after 2–3 days of culture.

### Viral infection of 3D and 2D intestinal organoids

For 3D organoid infection, the organoids were collected, and the Matrigel was removed by pipetting with PBS and centrifugation (200 × *g*, 5 min). The organoids were then exposed to TGEV (MOI = 5) for 1 h at 37 ℃. Subsequently, the organoids were washed three times with PBS and cultured in a Matrigel dome. TGEV was titrated at the indicated time points. For infections in 2D organoid monolayers, the organoid monolayers were washed with PBS and incubated with TGEV (MOI = 5) for 1 h at 37 ℃. The organoid monolayers were then washed three times with PBS, and the OGM was replaced. The culture supernatant was collected for TGEV titration, and the total RNA of the monolayers was extracted for detecting viral RNA copies for the indicated times. To analyse the anti-TGEV effect of TG, 3D or 2D organoids were pretreated or treated with TG at the indicated concentrations. The control group was exposed to the same volume of DMSO (Solarbio, Beijing, China). For EC_50_ assessments, TGEV RNA copy numbers in virus-infected 2D organoids were measured. The control group was used for normalization. The EC_50_ values were calculated by nonlinear regression analysis using GraphPad Prism 9.4.1.

### Cell viability assays

To assess the cytotoxicity of TG, cells and 2D organoid monolayers were treated with TG at the indicated concentrations or DMSO for 72 h. Cell viability was determined using a CCK-8 Cell Proliferation and Cytotoxicity Assay Kit (Solarbio) according to the manufacturer’s instructions. CC_50_ values were calculated by nonlinear regression analysis using GraphPad Prism 9.4.1.

### RT-qPCR

Total RNA was prepared using the TransZol Up Plus RNA Kit (TransGen, Beijing, China), and cDNA was synthesized using the HiScript II 1st Strand cDNA Synthesis Kit with gDNA wiper (Vazyme, Nanjing, China). The ChamQ SYBR qPCR Master Mix (Vazyme) was used to determine the relative mRNA levels of the genes. AceQ qPCR Probe Master Mix (Vazyme) was used to detect viral RNA copies. The primer and probe sequences used for the qPCR analysis are listed in Table 1.

**Table 1:** **Primers or probes used in this study**

Target gene	Primer/probe	Sequence (5ʹ-3ʹ)
TGEV N	Forward	TGCCATGAACAAACCAAC
	Reverse	GGCACTTTACCATCGAAT
	Probe	HEX-TAGCACCACGACTACCAAGC-BHQ1
TNF-α	Forward	GTCTCAAACCTCAGATAAG
	Reverse	GTTGTCTTTCAGCTTCAC
IFN-λ3	Forward	GCCAAGGATGCCTTTGAAGAG
	Reverse	CAGGACGCTGAGGGTCAGG
ISG56	Forward	AAATGAATGAAGCCCTGGAGTATT
	Reverse	AGGGATCAAGTCCCACAGATTTT
IL-6	Forward	AATGCTCTTCACCTCTCC
	Reverse	TCACACTTCTCATACTTCTCA
GAPDH	Forward	GGAAAGGCCATCACCATCTT
	Reverse	CATGGTCGTGAAGACACCAG
IFN-α	Forward	CTGCTGCCTGGAATGAGAGCC
	Reverse	TGACACAGGCTTCCAGGTCCC
IFN-λ1	Forward	CCACGTCGAACTTCAGGCTT
	Reverse	ATGTGCAAGTCTCCACTGGT
IL-1β	Forward	AATGCTCTTCACCTCTCC
	Reverse	TCACACTTCTCATACTTCTCA
IFN-β	Forward	CCACCACAGCTCTTTCCATGA
	Reverse	TGAGGAGTCCCAGGCAACT

### Western blotting

Total proteins were prepared from IPEC-J2 or Vero E6 cell samples using RIPA lysis buffer (Beyotime, Shanghai, China) supplemented with a protease inhibitor. A total of 20 µg of protein was separated by SDS-PAGE and transferred to PVDF membranes (Merck). The membranes were blocked with 5% skim milk (Solarbio) at room temperature for 2 h and immunoblotted overnight at 4 °C with primary antibodies against BiP (1:1000, Abcam, Waltham, USA), GAPDH (1:5000, Absin, Shanghai, China) and a laboratory-made monoclonal antibody against TGEV N or PEDV N (1:1000). A goat anti-Rab IgG secondary rabbit antibody (Absin) was diluted at 1:5000 for incubation with the membrane (at room temperature for 1 h). Finally, the immunoblots were developed with WesternBright ECL HRP substrate (Advansta, San Jose, USA) and imaged with a GelDoc XR (Bio-Rad, Hercules, USA).

### RNA-seq and bioinformatics

2D porcine intestinal organoids were infected with TGEV (MOI = 5). Then, mock- and TGEV-infected organoid monolayers were cultured in OGM supplemented with 0.5 μM TG. The same volume of DMSO was used as a control. The cultures were maintained for another 24 h. RNA was isolated from each group (three biological replicates). RNA sequencing was performed on an Illumina NovaSeq 6000 instrument by Gene Denovo Biotechnology Co., Ltd. (Guangzhou, China). The raw sequencing data can be available from the BioProject database (accession number: PRJNA1062093).

### Indirect immunofluorescence assay

Cells or 2D organoid monolayers were seeded and cultured on coverslips in 24-well cell culture plates. The cells or 2D organoids were infected with TGEV at the indicated MOIs and treated with TG. After 24 or 48 h, the cells and 2D organoids were fixed with 4% paraformaldehyde (Solarbio) for 15 min. After being permeabilized with 0.1% Triton X-100 (Solarbio) and blocked with 5% bovine serum albumin (Solarbio), the cells or 2D organoids were incubated with a primary antibody (a lab-made monoclonal antibody against TGEV N) and a fluorescence-labelled secondary antibody (Abcam). Then, the cells or 2D organoids were stained with DAPI (Solarbio) for 5 min and imaged with a confocal immunofluorescence microscope (LSM 900, Carl Zeiss, Jena, Germany).

### Histological analysis

Porcine small intestine tissue samples were fixed in formalin (Solarbio) and then dehydrated according to the standard protocol. The tissues were then embedded in paraffin. The tissue embedding blocks were sectioned and deparaffinized in xylene before being stained with haematoxylin and eosin (H&E). A double-blinded histological analysis was performed to evaluate the ratio of villus height to crypt depth. Ten complete villi from each section were randomly selected for analysis.

### Statistical analysis

All results are representative of three independent experiments. The data are presented as the mean ± standard error of the mean (SEM) and were analysed with two-tailed Student’s *t* tests or one-way analysis of variance using GraphPad Prism 9.4.1 (GraphPad Software, USA). *P* values < 0.05 were considered to indicate statistical significance and are indicated as * *p* < 0.05, ** *p* < 0.01, and *** *p* < 0.001. Pathway enrichment analyses (Figures 4B and C, 5B) were performed online via Metascape software.

## Results

### TG inhibits TGEV replication in IPEC-J2 cells

**Figure 1:**
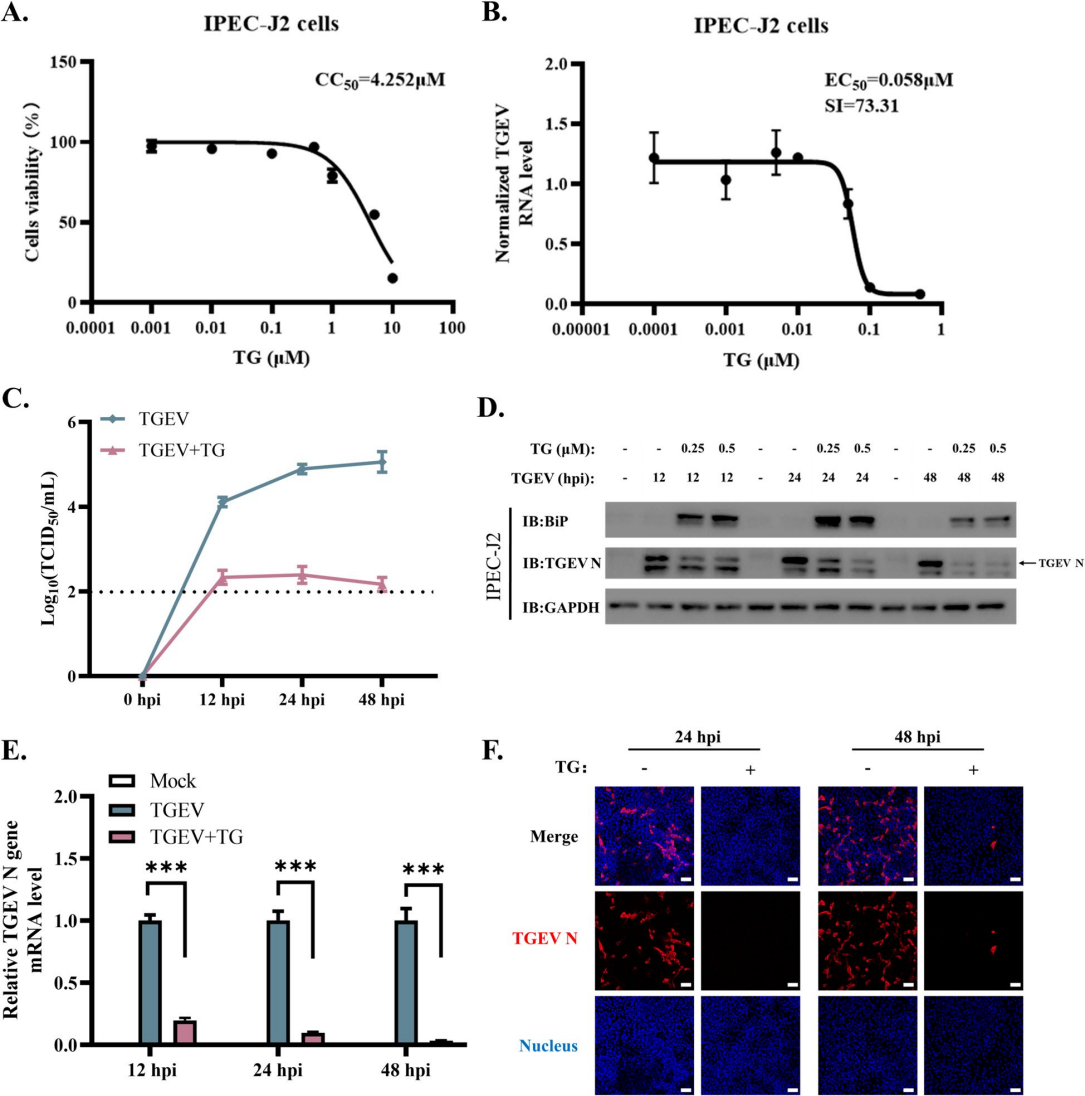
**TG inhibits TGEV replication in IPEC-J2 cells.** **A**, **B** Assessment of the half-maximal effective concentration (EC_50_) and cytotoxic concentration (CC_50_) of TG in IPEC-J2 cells. **C** The supernatant of IPEC-J2 cells at 0, 12, 24, and 48 hpi (MOI = 1) was harvested to titrate TGEV using the TCID_50_ assay. The detection limit is shown as a dotted line. **D** Total protein was extracted from IPEC-J2 cells to detect the expression of TGEV N and BiP. **E** The RNA of IPEC-J2 cells was isolated for testing TGEV viral RNA copies. **F** TGEV-infected IPEC-J2 cells (MOI = 1) in the TG or DMSO group were fixed to image TGEV-infected cells (scale bar = 50 μm). All experiments were performed in triplicate. *P* values < 0.05 were considered to indicate statistical significance and are indicated as *** *p* < 0.001.

To evaluate the effects of TG on TGEV infection in intestinal epithelial cells, the half-maximal effective and cytotoxic concentrations (EC_50_ and CC_50_) of TG were assessed in TGEV-infected (MOI = 1) or mock-infected IPEC-J2 cells. At 48 h post-TG treatment, the cell viability of IPEC-J2 cells decreased in a dose-dependent manner, with a CC_50_ of 4.252 µM (Figure 1A). In TGEV-infected IPEC-J2 cells, TG attenuated TGEV replication with an EC_50_ of 0.058 µM at 48 h post-infection (hpi) (Figure 1B). TG exhibited a selectivity index (SI: CC_50_/EC_50_) of 73.31 for inhibiting TGEV in IPEC-J2 cells, which suggested an acceptable safety margin. In ST (swine testicle) cells, which are susceptible to TGEV, TG had a CC_50_ of 3.152 µM, an EC_50_ of 0.002 µM, and an SI of 1576 (Additional file 1A and B). TG exhibited a distinct safety margin in inhibiting TGEV infection in both TGEV-susceptible cells and intestinal epithelial cells. To further investigate the activity of TG against TGEV, the replication dynamics of TGEV in IPEC-J2 cells were detected after TG treatment. After TGEV infection, IPEC-J2 cells were continuously exposed to 0.5 µM TG for 2 days. The results demonstrated that TG significantly decreased the TGEV viral titre in the culture supernatant and the viral RNA copy number in the cell pellets at 12, 24, and 48 hpi (Figure 1C and E). Moreover, TG treatment induced the expression of BiP (Figure 1D), a chaperone protein of ERS, and inhibited TGEV replication (Figure 1D and E). Additionally, an indirect immunofluorescence assay (IFA) also showed that TG significantly attenuated TGEV propagation (Figure 1F). These data demonstrate that TG inhibits TGEV replication in IPEC-J2 cells.

To determine the potential of TG to inhibit SeCoVs, we investigated the effect of TG on PEDV, another widely spreading SeCoV. Excitingly, TG had a CC_50_ of 6.816 µM, an EC_50_ of 0.147 µM, and an SI of 46.37, suggesting that TG is a safe margin for preventing PEDV replication in Vero E6 cells (Additional file 2A and B). Moreover, 0.25 µM TG decreased the PEDV viral titre in culture supernatants and the number of RNA copies in Vero E6 cells at 12, 24, and 48 hpi (Additional file 2C and D). Western blot results showed that TG stimulated the expression of BiP and inhibited the expression of the PEDV N protein (Additional file 2E). Taken together, these results suggested that TG is a broad-spectrum antiviral agent against SeCoVs.

TG suppresses the replication of tick-borne encephalitis virus (TBEV) by stimulating interferon (IFN) responses [17]. We next sought to investigate the effect of TG on IFN responses in TGEV-infected IPEC-J2 cells. The transcriptional levels of the main IFNs, including IFN-α (*Ifna*), IFN-β (*Ifnb*), IFN-λ1 (*Ifnl1*) and IFN-λ3 (*Ifnl3*), were tested by RT-qPCR. The results showed that TGEV infection upregulated the mRNA levels of these four IFNs, and TG treatment decreased the expression of these IFNs, especially IFN-β, IFN-λ1, and IFN-λ3 (Additional file 3A–D). The IFN-stimulated genes OASL (*Oasl*) and ISG56 were also inhibited by TG (Additional file 3E and F). These data suggested that TG inhibited TGEV replication in an IFN-independent manner. Additionally, the transcription of interleukin 6 (IL-6) and interleukin-1β (IL-1β) was significantly promoted by TG treatment (Additional file 3G–I). These results suggest that TG has a potential risk of promoting inflammatory responses.

### TG suppresses TGEV replication in 2D intestinal organoid monolayers

**Figure 2:**
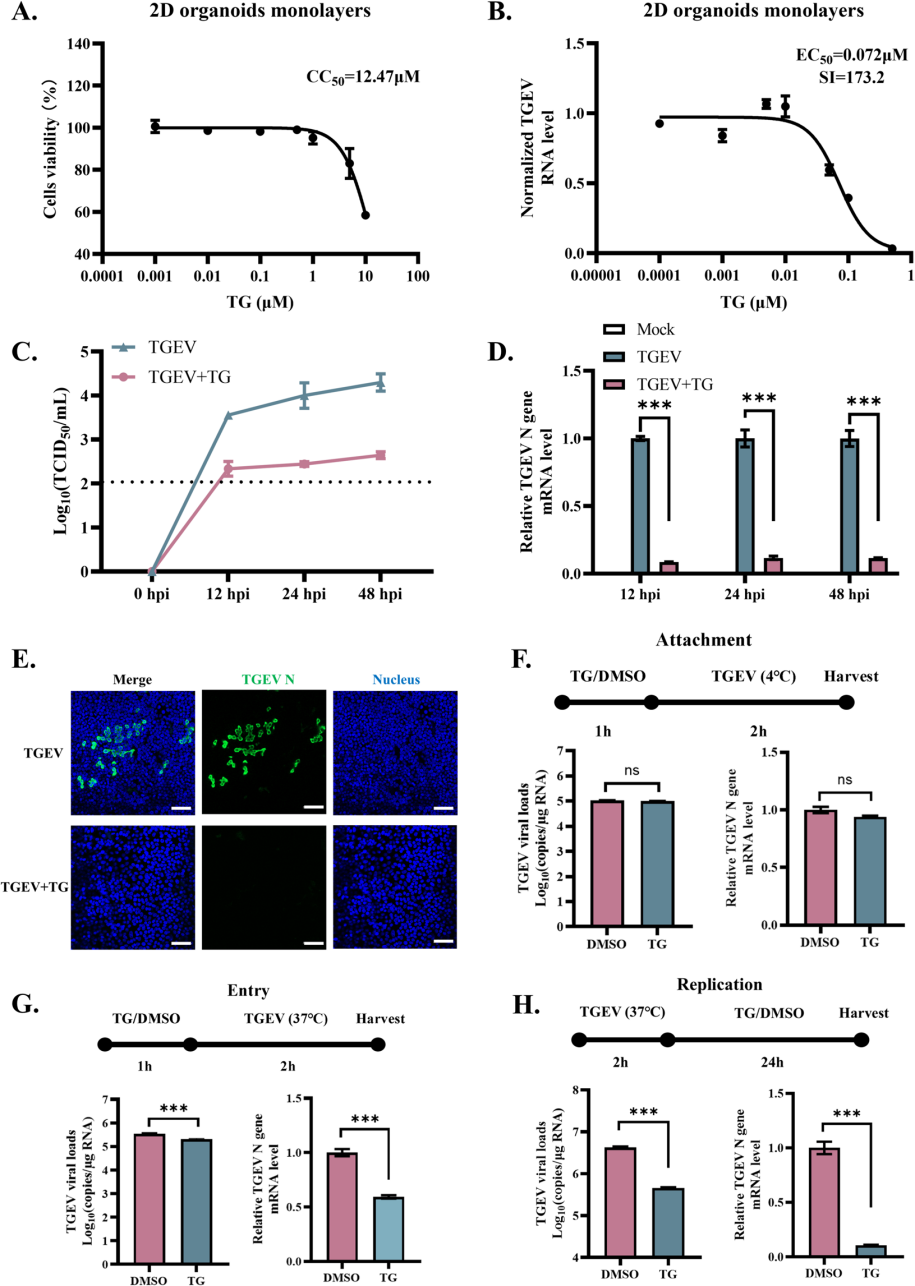
**TG suppresses TGEV replication in 2D intestinal organoid monolayers.** **A**, **B** Assessment of the half-maximal effective concentration (EC_50_) and cytotoxic concentration (CC_50_) of TG in TGEV-infected and noninfected 2D intestinal organoid monolayers. **C** At 0, 12, 24, and 48 hpi (MOI = 5), TGEV titres in organoid monolayer culture supernatants were determined by TCID_50_ assay. The limit of detection is shown as a dotted line. **D** TGEV RNA copy numbers in epithelial cells were quantified at 12, 24, and 48 hpi. **E** Indirect immunofluorescence assay (IFA) was performed to determine the reduction in TGEV-infected cells in organoid monolayers induced by TG (scale bar = 50 μm). **F**–**H** The effects of TG on TGEV attachment, internalization, and replication were investigated. All experiments were performed in triplicate. *P* values < 0.05 were considered to indicate statistical significance and are indicated as *** *p* < 0.001.

The antiviral effects of TG against TGEV in cell lines have been previously described. However, the complexity and rapid renewal of the intestinal epithelium necessitate the use of more representative models. Intestinal organoids provide a ground-breaking solution for mimicking this complex tissue. In previous studies, mature 3D and 2D porcine intestinal organoid cultures were established [14, 15]. To further investigate the antiviral potential of TG against enteric coronavirus, 2D porcine intestinal organoid monolayers were used as a novel platform. The EC_50_ and CC_50_ of TG against TGEV-infected (MOI = 5) or mock-infected 2D intestinal organoid monolayers were assessed. At 48 h post-TG treatment, the viability of epithelial cells decreased in a dose-dependent manner, with a CC_50_ of 12.47 µM (Figure 2A). In organoid monolayers, TG had an EC_50_ of 0.072 µM and an SI of 173.2, which suggested that TG had a better safety margin than in IPEC-J2 cells (Figure 2B). TG at 0.5 µM decreased the TGEV viral titre in organoid monolayer culture supernatants and the number of TGEV RNA copies in epithelial cells at 12, 24, and 48 hpi (Figure 2C and D). The IFA results demonstrated that TG at 0.5 µM significantly reduced the number of TGEV-positive cells in organoid monolayers compared to that in the DMSO control group (Figure 2E).

We also evaluated the cytotoxicity and anti-TGEV effect of TG in 3D porcine intestinal organoids. The organoids were treated with TG for three days. Treatment with 0.5 µM TG induced massive apoptosis in the organoids at 3 days post-treatment (Additional file 4A). A concentration of 0.25 µM TG reduced the TGEV titre in the organoid culture supernatant and significantly decreased the TGEV-positive cell number in the organoids (Additional file 4B and 4C).

Given the notable inhibitory effect of TG on TGEV replication, we next assessed whether TG also affects the attachment and internalization of TGEV in organoid monolayers. After one hour of TG treatment, the organoid monolayers were incubated with TGEV (MOI = 5) for 2 h. To investigate TGEV attachment, the organoid monolayers were incubated with TGEV at 4 °C, while they were incubated at 37 °C to test viral internalization. Before RNA isolation, the organoid monolayers were washed with citrate buffer to remove any unattached TGEV. RT-qPCR revealed that TG pretreatment did not affect TGEV attachment to the organoid monolayers (Figure 2F). However, TG significantly diminished TGEV internalization in the organoid monolayers (Figure 2G). The effect of TG intervention on TGEV replication was also tested. Consistent with the studies above, TG intervention impaired TGEV replication in the organoid monolayers (Figure 2H). Collectively, these results suggest that TG suppresses TGEV replication in an organoid culture model.

### TG promotes the expression of ERAD components in 2D intestinal organoid monolayers

**Figure 3:**
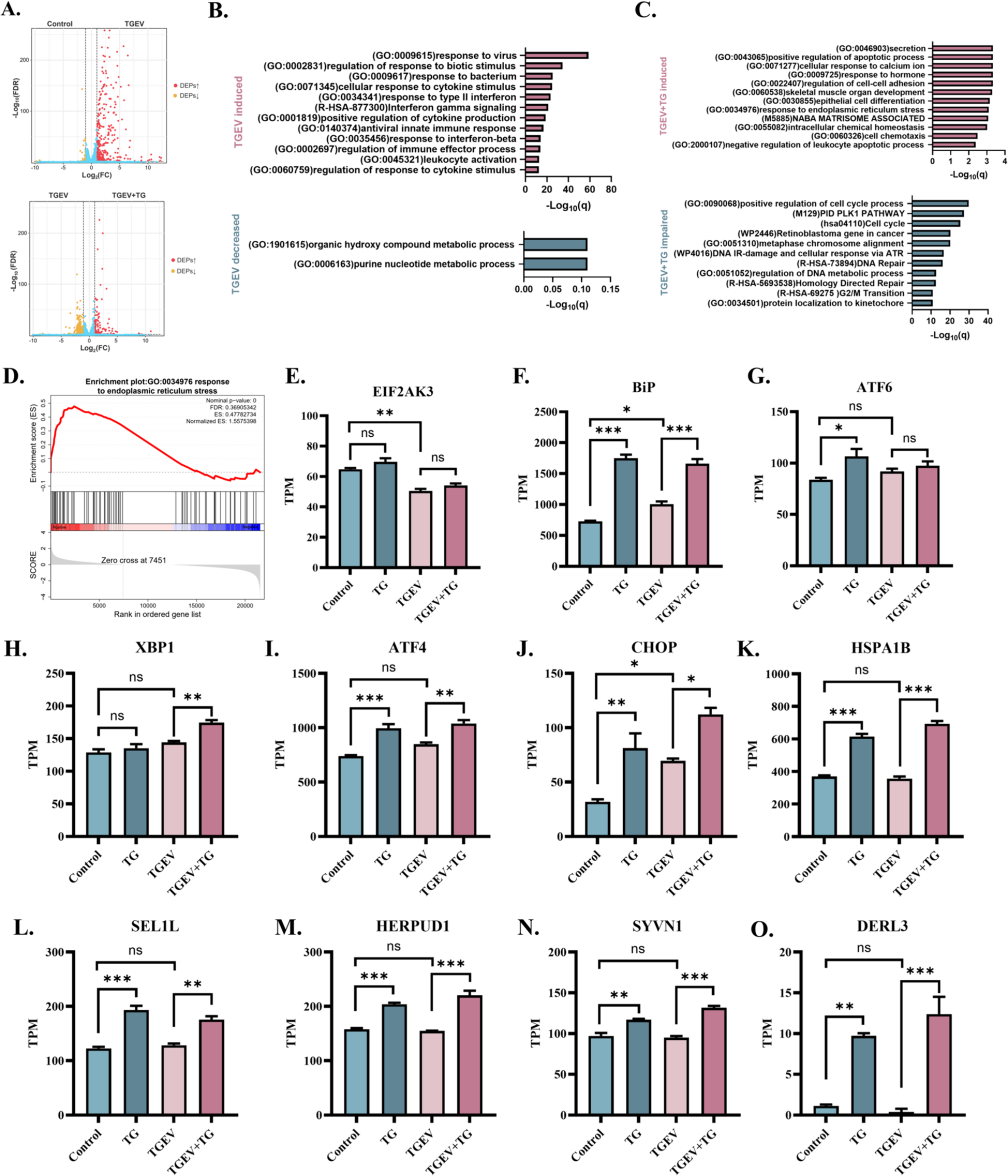
**TG promotes the expression of ERAD components in 2D intestinal organoid monolayers.** **A** RNA-seq-identified DEGs induced by TGEV infection and TG intervention (volcano plot). **B** ORA of DEGs induced and suppressed by TGEV infection in 2D intestinal organoid monolayers based on Metascape software. **C** ORA of DEGs induced and suppressed by TG intervention in TGEV-infected 2D intestinal organoid monolayers based on Metascape software. **D** Gene set enrichment analysis (GSEA) of TG-induced DEGs in TGEV-infected 2D intestinal organoids. **E**–**J** The expression levels of ERS-associated genes in each group. **K**–**O** The expression levels of ERAD components, including HSPA1B, SEL1L, HERPUD1, SYVN1 and DERL3. Gene expression data were calculated as transcripts per million reads (TPM). All experiments were performed in triplicate. *P* values < 0.05 were considered to indicate statistical significance and are indicated as * *p* < 0.05, ** *p* < 0.01, and *** *p* < 0.001.

To characterize the underlying mechanism by which TG suppresses TGEV infection in intestinal organoid monolayers, we performed RNA-seq to identify the differentially expressed genes (DEGs) induced by TGEV infection and TG intervention. Compared with control organoid monolayers, TGEV-infected organoids exhibited 39 downregulated genes and 369 upregulated genes at 24 hpi (Figure 3A). In TGEV-infected organoid monolayers, TG treatment increased the mRNA levels of 140 genes and suppressed the expression of 304 genes (Figure 3A). To link these DEGs to specific biological functions, Metascape software was used for overrepresentation analysis (ORA) [18]. As expected, TGEV mainly induced the transcription of genes related to antiviral responses, IFN responses, cytokine production, and cytokine stimulation in organoid monolayers. TGEV also suppressed the expression of mRNAs associated with organic hydroxy compound and purine nucleotide metabolic processes (Figure 3B). In TGEV-infected organoid monolayers, TG induced the expression of mRNAs related to secretion, the positive regulation of apoptotic processes, cellular responses to calcium ions and hormones, responses to ERS, and cell chemotaxis. TG intervention mainly decreased the transcription of genes related to the cell cycle and DNA repair in TGEV-infected organoid monolayers (Figure 3C).

Previous investigations reported that TG inhibits coronavirus by affecting several central mechanisms required for coronavirus replication, including ER quality control (ERQC), ER-associated protein degradation (ERAD), and selective autophagic flux [8]. Therefore, we further analysed the effect of TG on TGEV-infected organoid monolayers. Gene Set Enrichment Analysis (GSEA) revealed that TG intervention promoted the response to ER stress in TGEV-infected organoid monolayers (Figure 3D). Furthermore, we assessed the expression of ER stress-related genes in the transcriptome data. TG did not affect the expression of EIF2AK3; however, TG stimulated the expression of BiP, ATF6, XBP1, ATF4, and CHOP in TGEV-infected or uninfected organoid monolayers (Figure 3E–J). Interestingly, TG promoted the transcription of important ERAD components, including HSPA1B, SEL1L, HERPUD1, SYVN1, and DERL3 (Figure 3K–O). Collectively, these data suggest that TG affects a range of important biological functions in organoid monolayers, including ER stress, ERAD, and metabolic processes. These changes imply underlying mechanisms by which TG affects the replication of TGEV.

### TG regulates intestinal epithelial cell differentiation

**Figure 4:**
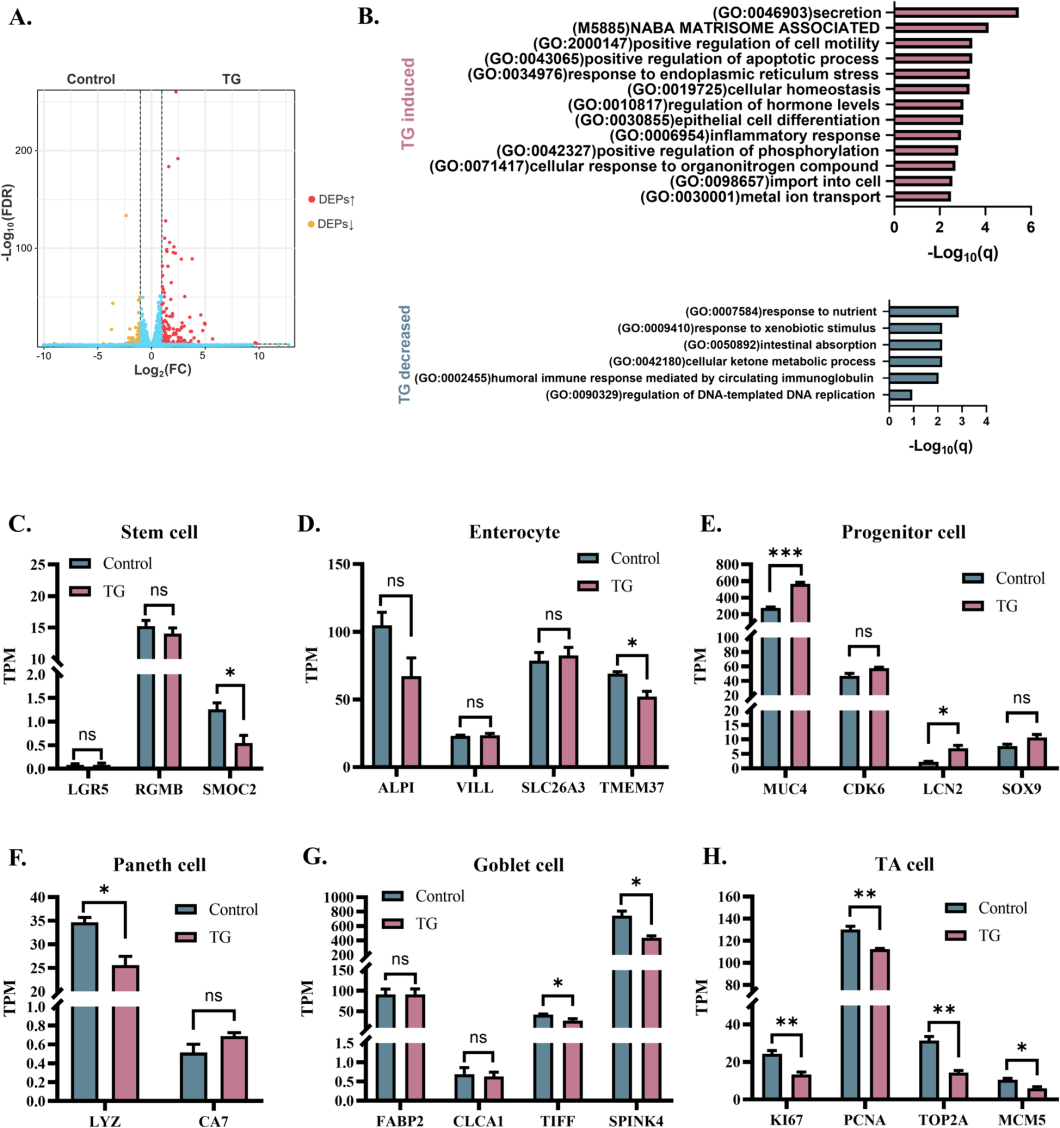
**TG regulates intestinal epithelial cell differentiation.** **A** RNA-seq identified DEGs induced by TG in intestinal organoid monolayers (volcano plot). **B** ORA of DEGs induced or suppressed by TG treatment in 2D intestinal organoid monolayers based on Metascape software. **C** The gene expression levels of intestinal stem cell markers in each group. **D** The gene expression levels of the enterocyte markers. **E** The expression levels of progenitor cell marker genes. **F** The expression levels of Paneth cell-specific genes. **G** The expression levels of goblet cell marker genes. **H** The expression levels of TA cell marker genes. Gene expression data were calculated as transcripts per million reads (TPM). All experiments were performed in triplicate. *P* values < 0.05 were considered to indicate statistical significance and are indicated as * *p* < 0.05 and ** *p* < 0.01.

**Figure 5:**
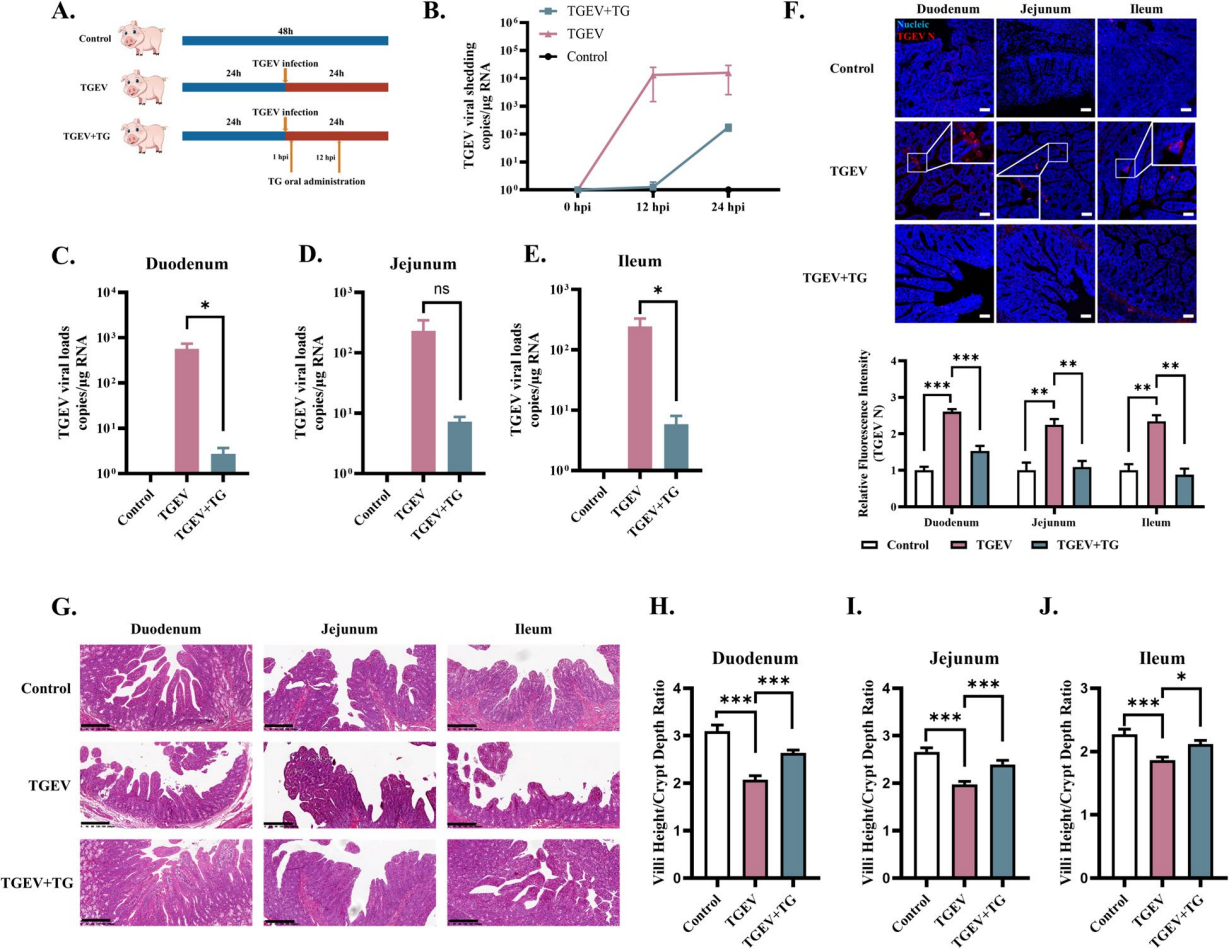
**Oral administration of TG suppresses TGEV infection in neonatal piglets.** **A** Neonatal piglets (*n* = 3) were orally challenged with TGEV at a dose of 3 × 10^7^ TCID_50_ per piglet. They were then orally administered TG at a dosage of 2 μg/kg at 1 and 12 hpi. Anal swabs were collected at 0, 12, and 24 hpi to assess TGEV shedding. **B** TGEV shedding at 12 and 24 hpi was tested using RT-qPCR. **C**–**E** At 24 hpi, the piglets were sacrificed, and TGEV viral loads in intestinal tissue were assessed using RT-qPCR. **F** TGEV-infected cells in the small intestines of piglets were detected through IFA (scale bar = 50 μm). **G** Haematoxylin–eosin (HE) staining was performed to assess the effect of TG intervention on the intestines of TGEV-infected piglets (scale bar = 200 μm). **H**–**J** Double-blinded histological analysis was performed to evaluate the villus height/crypt depth ratio as a measure of TGEV-induced pathological injury of the small intestine. *P* values < 0.05 were considered to indicate statistical significance and are indicated as * *p* < 0.05 and *** *p* < 0.001.

Intestinal epithelial homeostasis is maintained by stem cells in the crypt that renew with differentiation into various mature cell types. Next, we analysed the effect of TG on the intestinal epithelium using RNA-seq. In the DMSO control versus TG treatment group intestinal organoid monolayers, 160 upregulated and 82 downregulated DEGs were identified (Figure 4A). ORA suggested that TG treatment promoted secretion, positive regulation of cell motility and apoptotic processes, response to ERS, cellular homeostasis, epithelial cell differentiation and inflammation (Figure 5B). The downregulated DEGs were mainly enriched in response to nutrients, intestinal absorption, and cellular ketone metabolic processes (Figure 4B). We further analysed the effect of TG on epithelial cell differentiation through the expression of cell subtype marker genes. TG slightly suppressed the mRNA level of SMOC2, an intestinal stem cell marker gene (Figure 4C). TG also did not have a significant impact on most of the marker genes of enterocytes, although TG restrained the transcription of TMEM37 (Figure 4D). TG significantly promoted the expression of two progenitor cell-related genes, MUC4 and LCN2 (Figure 4E). TG suppressed the mRNA levels of the Paneth cell feature gene (LYZ) and two of the goblet cell marker genes (TIFF and SPINK4) (Figure 4F and G). In addition, TG significantly decreased the mRNA levels of transit-amplifying cell (TA cell) marker genes, including KI67, PCNA, TOP2A, and MCM5 (Figure 4H). TA cells develop from crypt stem cells, which can further differentiate into absorptive or secretory epithelial cells. The impairment of TA cells may affect intestinal epithelial homeostasis. These data suggest that TG regulates intestinal epithelial cell development at the transcriptional level.

### Oral administration of TG suppresses TGEV infection in neonatal piglets

The potential of TG as an oral antiviral agent against TGEV infection was evaluated. Neonatal piglets were orally challenged with TGEV (3 × 10^7^ TCID_50_ for each piglet), followed by oral TG administration (2 μg/kg) at 1 and 12 hpi, as depicted in the illustration (Figure 5A). Anal swabs were collected to test for TGEV shedding at 0, 12, and 24 hpi. The results showed that oral administration of TG significantly suppressed viral shedding at 12 and 24 hpi (Figure 5B). At 24 hpi, the piglets were sacrificed, and viral loads in the intestinal tissue were assessed by RT-qPCR. Compared to TGEV-infected piglets, TG-treated piglets had fewer TGEV RNA copies in the duodenum, jejunum, and ileum (Figure 5C–E). Next, we detected TGEV-infected cells in the small intestines of piglets through IFA. The results showed that oral TG administration significantly decreased the number of TGEV-positive cells in the small intestine (Figure 5F). These data illustrate that the oral administration of TG suppressed TGEV infection in neonatal piglets.

SeCoV infection typically triggers inflammatory responses and pathological changes in the intestines, which are characterized by inflammatory cell infiltration and intestinal villus atrophy. Haematoxylin–eosin (HE) staining was performed to assess the effect of TG intervention on the intestines of TGEV-infected piglets. The results showed that oral TG administration relieves inflammatory cell infiltration caused by TGEV infection (Figure 5G). The villus height/crypt depth ratio was calculated to assess TGEV-induced pathological injury to the small intestine. TG intervention significantly reversed the decrease in the villus height/crypt depth ratio caused by TGEV infection in the piglet small intestine (Figure 5H–J). To evaluate the hepatorenal toxicity of TG in piglets, kidney (CREA) and liver (AST, ALT, ALB) functional parameters were measured from collected serum samples. The results demonstrated that oral administration of TG did not change the physiological levels of CREA, AST, ALT, or ALB in the serum (Additional file 5A–D), suggesting that short-term oral administration of TG does not noticeably affect the hepatic or renal function of piglets. Collectively, these data suggest that TG relieves TGEV-induced pathological injury in the small intestine.

## Discussion

Vaccines are commonly used to prevent viral infectious diseases but lack protection for mutant and emerging viruses. Thus, the development of antiviral drugs is necessary. Previous investigations demonstrated the ability of TG to suppress human coronavirus, influenza virus, and respiratory syncytial virus. In this study, we systematically assessed the potential of TG as an oral antiviral against SeCoVs. Initially, TG inhibited TGEV in porcine intestinal epithelial cells (IPEC-J2 cells) and intestinal organoids. Additionally, TG effectively restrained TGEV infection in neonatal piglets. RNA-seq revealed that TG activated ERS and promoted the expression of ERAD components, including HSPA1B, SEL1L, HERPUD1, SYVN1, and DERL3, in both TGEV-infected and noninfected 2D organoids. These findings align with previous studies and indicate that TG promoted the expression of core ERAD components, including HERPUD1, CAPN2, OS9, and SEC61B [8]. ERAD is a degradative mechanism of misfolded proteins activated by the UPR. The crosstalk between ERAD and coronavirus replication remains unclear. HSPA1B is considered to mediate the inhibitory effect of Orf virus on goat skin fibroblasts [19]. SEL1L inhibits hepatitis B virus in an ERAD- and ERQC-autophagy-dependent manner [20]. The link between host HSPA1B, SEL1L, and coronavirus replication remains unestablished. HERPUD1 regulates host MAVS signalling and promotes the phosphorylation and nuclear translocation of IRF3 during RNA virus infection [21]. In this study, our results showed that TG suppresses TGEV replication but not through the IFN pathway. Our additional evidence suggested that TG restrains TGEV replication potentially through activating ERAD and ERAD-related molecules, but the detailed underlying mechanism still needs to be elucidated.

The homeostasis of the intestinal epithelium is important for maintaining its physical barrier function. The potential function of TG as an oral antiviral for epithelial differentiation cannot be ignored. Intestinal organoids provide a physiological platform for further investigating the effects of TG on intestinal epithelial differentiation. Through RNA-seq, we observed that TG altered intestinal epithelial differentiation at the transcriptional level. Specifically, oral administration of TG inhibited the expression of genes associated with Paneth cells, goblet cells, and TA cells while enhancing the mRNA levels of progenitor cell marker genes. Paneth cells produce granules containing antimicrobial peptides that are crucial for gut defence, while goblet cells secrete a mucus layer that separates the intestinal epithelium from the luminal microbial content. Progenitor and TA cells are derived from intestinal stem cells, which commit to mature intestinal epithelial cell lineages. Our data suggested that TG suppressed the development of Paneth cells, goblet cells, and TA cells while promoting progenitor cell production. These findings raise concerns about the potential side effects of TG on the gastrointestinal mucosa. In vivo studies have shown that TG intervention partly facilitates the repair of pathological damage caused by TGEV infection. However, further long-term studies are necessary to determine the effect of TG on intestinal epithelial differentiation and mucosal barrier function.

In summary, our comprehensive research demonstrated the effective anti-TGEV properties of TG in multiple models. Our findings underscore the potential clinical utility of TG for controlling enteric coronavirus infections. However, further investigation is required to elucidate the precise mechanism by which TG suppresses viral replication, and additional research is necessary to evaluate the short-term clinical therapeutic applications of TG.

### Supplementary Information

**Additional file 1. TG inhibits TGEV replication in ST cells.** (A-B) Assessment of the half-maximal effectiveness (EC_50_) and cytotoxicity (CC_50_) of TG in TGEV-infected (MOI = 0.1) or noninfected ST cells.

**Additional file 2. TG inhibits PEDV replication in Vero cells.** (A-B) Assessment of the half-maximal effective concentration (EC_50_) and cytotoxic concentration (CC_50_) of TG in PEDV-infected (MOI = 0.1) or noninfected Vero cells. (C) RNA was isolated from Vero cells to test for PEDV viral RNA copies. (D) Supernatants from Vero cells at 0, 12, 24, and 48 hpi (MOI = 0.1) were harvested to titrate PEDV using a TCID_50_ assay. The detection limit is shown as a dotted line. (E) Total proteins were prepared from Vero cells to detect the expression of PEDV N and BiP. All experiments were performed in triplicate. *P* values < 0.05 were considered to indicate statistical significance and are indicated as * *p* < 0.05 and ** *p* < 0.01.

**Additional file 3. TG impairs the IFN response of IPEC-J2 cells upon TGEV infection.** (A-D) IPEC-J2 cells at 0, 12, 24, and 48 hpi (MOI = 1) were harvested to extract total RNA. The transcriptional levels of the main IFNs, including IFN-α (*Ifna*), IFN-β (*Ifnb*), IFN-λ1 (*Ifnl1*), and IFN-λ3 (*Ifnl3*), were tested by RT-qPCR. (E–F) The transcription of two IFN-stimulated genes, OASL (*Oasl*) and ISG56 (*Isg56*), was detected. (G-I) The transcription of IL-6, IL-1β, and TNF-α was tested by RT-qPCR. The RT-qPCR data were calculated using the comparative threshold cycle (2^−ΔΔCT^) method. All experiments were performed in triplicate. *P* values < 0.05 were considered to indicate statistical significance and are indicated as * *p* < 0.05, ** *p* < 0.01, and *** *p* < 0.001.

**Additional file 4. TG inhibits TGEV replication in 3D organoids.** (A) 3D organoids were treated with TG at the indicated concentrations for three days. Organoids were imaged using bright-field microscopy (scale bar = 50 μm). (B) The supernatant of 3D organoids was harvested at 0, 12, 24, and 48 hpi (MOI = 5) to titrate TGEV using a TCID_50_ assay. The detection limit is shown as a dotted line. (C) IFA was performed to determine the impact of 0.25 μM TG on the reduction in TGEV-infected cells in 3D organoids (scale bar = 50 μm). All experiments were performed in triplicate.

**Additional file 5. Short-term oral administration of TG does not cause liver or kidney toxicity in piglets.** Neonatal piglets (*n* = 3) were orally challenged with TGEV at a dose of 3 × 10^7^ TCID_50_ per piglet. They were then orally administered TG at a dosage of 2 μg/kg at 1 and 12 hpi. Serum samples were collected at 24 hpi after the animals were sacrificed. The levels of serum CREA (A), AST (B), ALT (C), and ALB (D) were detected to evaluate TG-induced liver and kidney toxicity in the piglets.

**Additional file 6. Immunostaining of TGEV N protein in the mock-infected group of IPEC-J2 cells and 2D intestinal organoid monolayers.** (A) IFA was performed to detect TGEV in IPEC-J2 cells (scale bar = 50 μm). (B) IFA was performed to detect TGEV-infected cells on 2D intestinal organoid monolayers (scale bar = 50 μm). All experiments were performed in triplicate.

## Data Availability

The data that support the findings of this study are available upon request from the corresponding author. The raw RNA-seq data are openly available in the BioProject database (accession number: PRJNA1062093). The remaining data generated in this study are provided in the additional files with this paper.
