# Supplementary figures and images for: In vitro and in vivo evaluation of thapsigargin as an antiviral agent against transmissible gastroenteritis virus
Source: Vet Res. 2024 Aug 2;55:97. doi: 10.1186/s13567-024-01359-x (PMC11297606; doi:10.1186/s13567-024-01359-x)

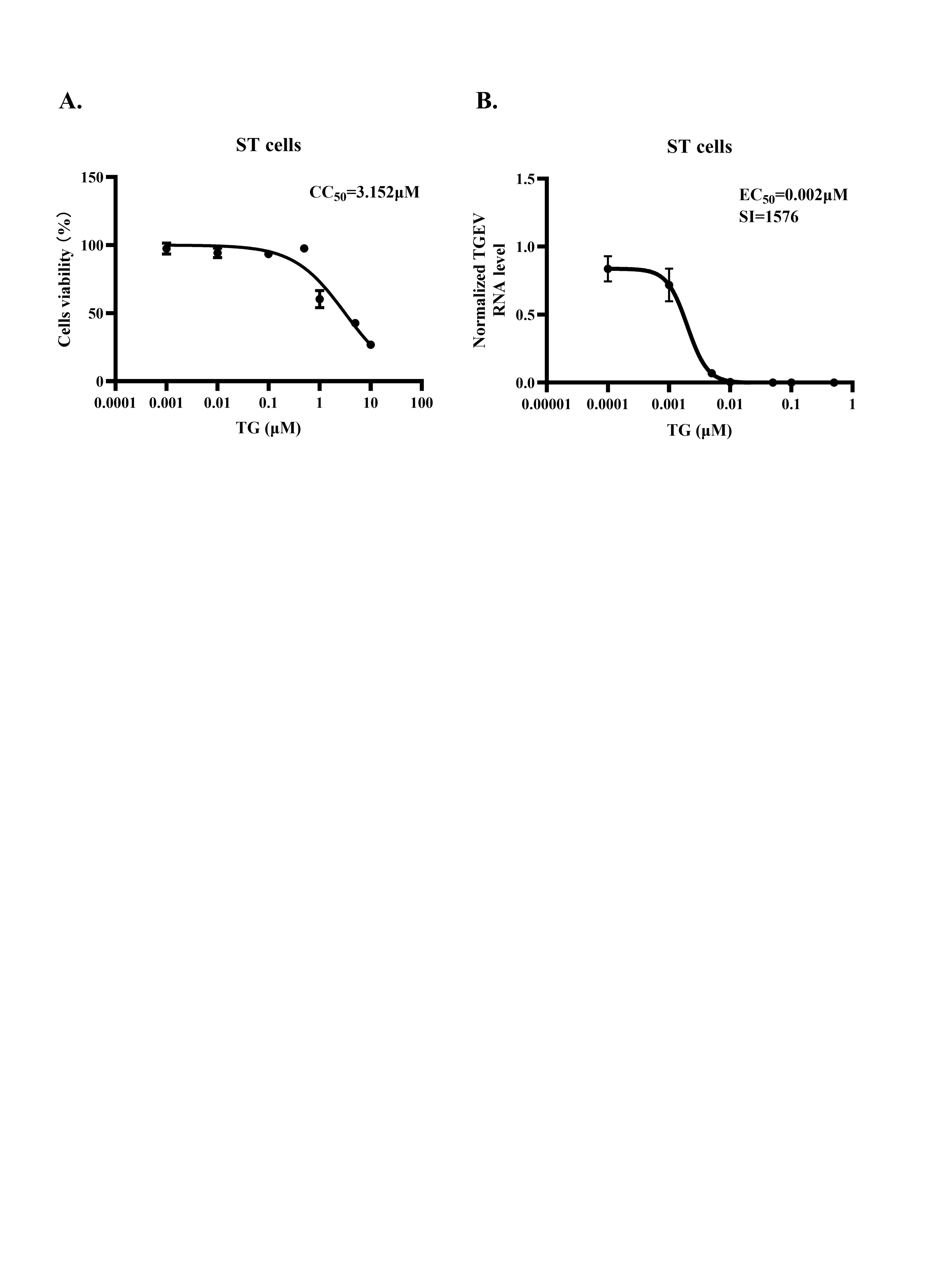

Supplement: Supplementary file 1 — Additional file 1. TG inhibits TGEV replication in ST cells. (A-B) Assessment of the half-maximal effectiveness (EC50) and cytotoxicity (CC50) of TG in TGEV-infected (MOI = 0.1) or noninfected ST cells. [file 13567_2024_1359_MOESM1_ESM.tif]

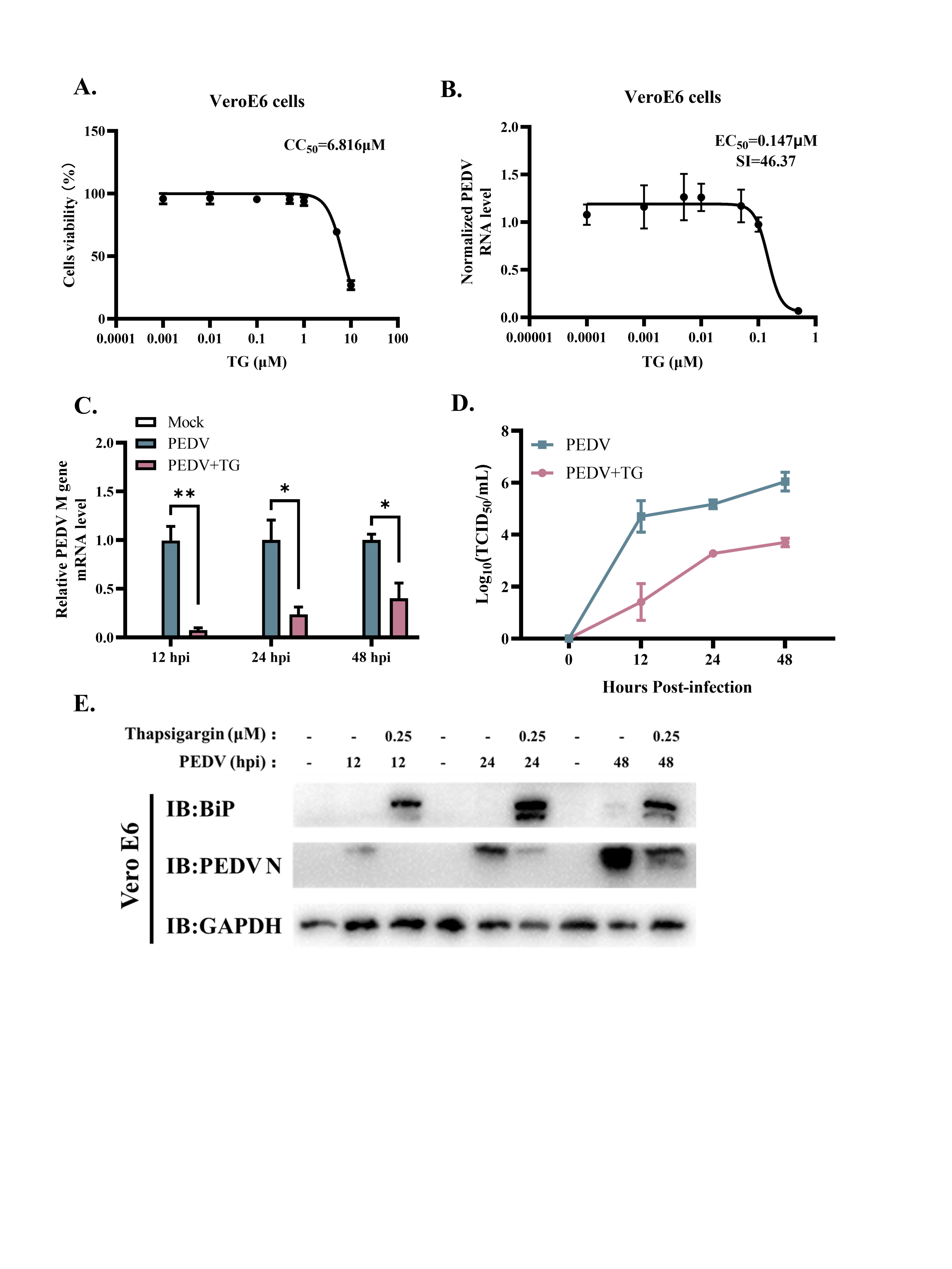

Supplement: Supplementary file 2 — Additional file 2. TG inhibits PEDV replication in Vero cells. (A-B) Assessment of the half-maximal effective concentration (EC50) and cytotoxic concentration (CC50) of TG in PEDV-infected (MOI = 0.1) or noninfected Vero cells. (C) RNA was isolated from Vero cells to test for PEDV viral RNA copies. (D) Supernatants from Vero cells at 0, 12, 24, and 48 hpi (MOI = 0.1) were harvested to titrate PEDV using a TCID50 assay. The detection limit is shown as a dotted line. (E) Total proteins were prepared from Vero cells to detect the expression of PEDV N and BiP. All experiments were performed in triplicate. P values < 0.05 were considered to indicate statistical significance and are indicated as * p < 0.05 and ** p < 0.01. [file 13567_2024_1359_MOESM2_ESM.tif]

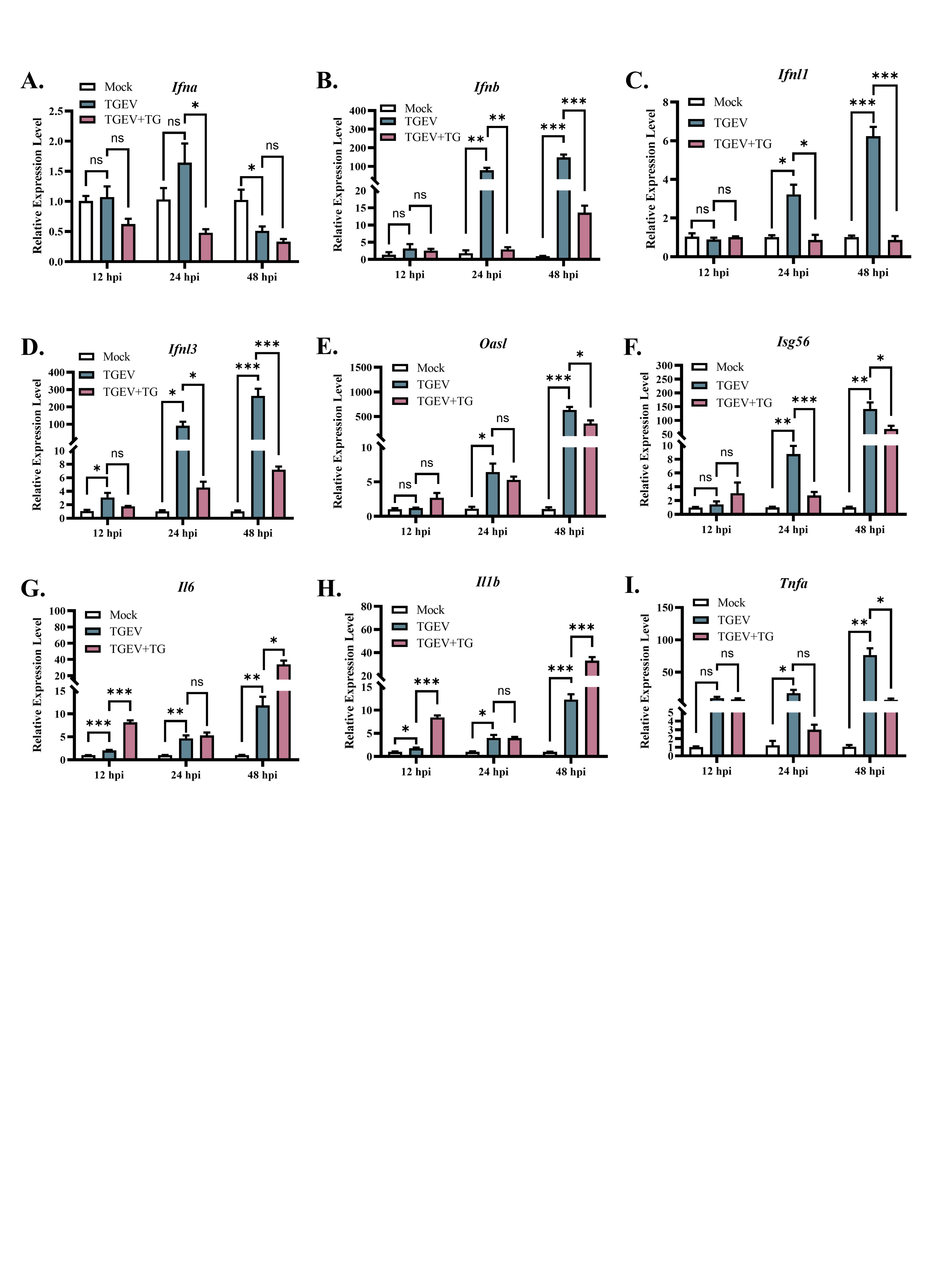

Supplement: Supplementary file 3 — Additional file 3. TG impairs the IFN response of IPEC-J2 cells upon TGEV infection. (A-D) IPEC-J2 cells at 0, 12, 24, and 48 hpi (MOI = 1) were harvested to extract total RNA. The transcriptional levels of the main IFNs, including IFN-α (Ifna), IFN-β (Ifnb), IFN-λ1 (Ifnl1), and IFN-λ3 (Ifnl3), were tested by RT-qPCR. (E–F) The transcription of two IFN-stimulated genes, OASL (Oasl) and ISG56 (Isg56), was detected. (G-I) The transcription of IL-6, IL-1β, and TNF-α was tested by RT-qPCR. The RT-qPCR data were calculated using the comparative threshold cycle (2−ΔΔCT) method. All experiments were performed in triplicate. P values < 0.05 were considered to indicate statistical significance and are indicated as * p < 0.05, ** p < 0.01, and *** p < 0.001. [file 13567_2024_1359_MOESM3_ESM.tif]

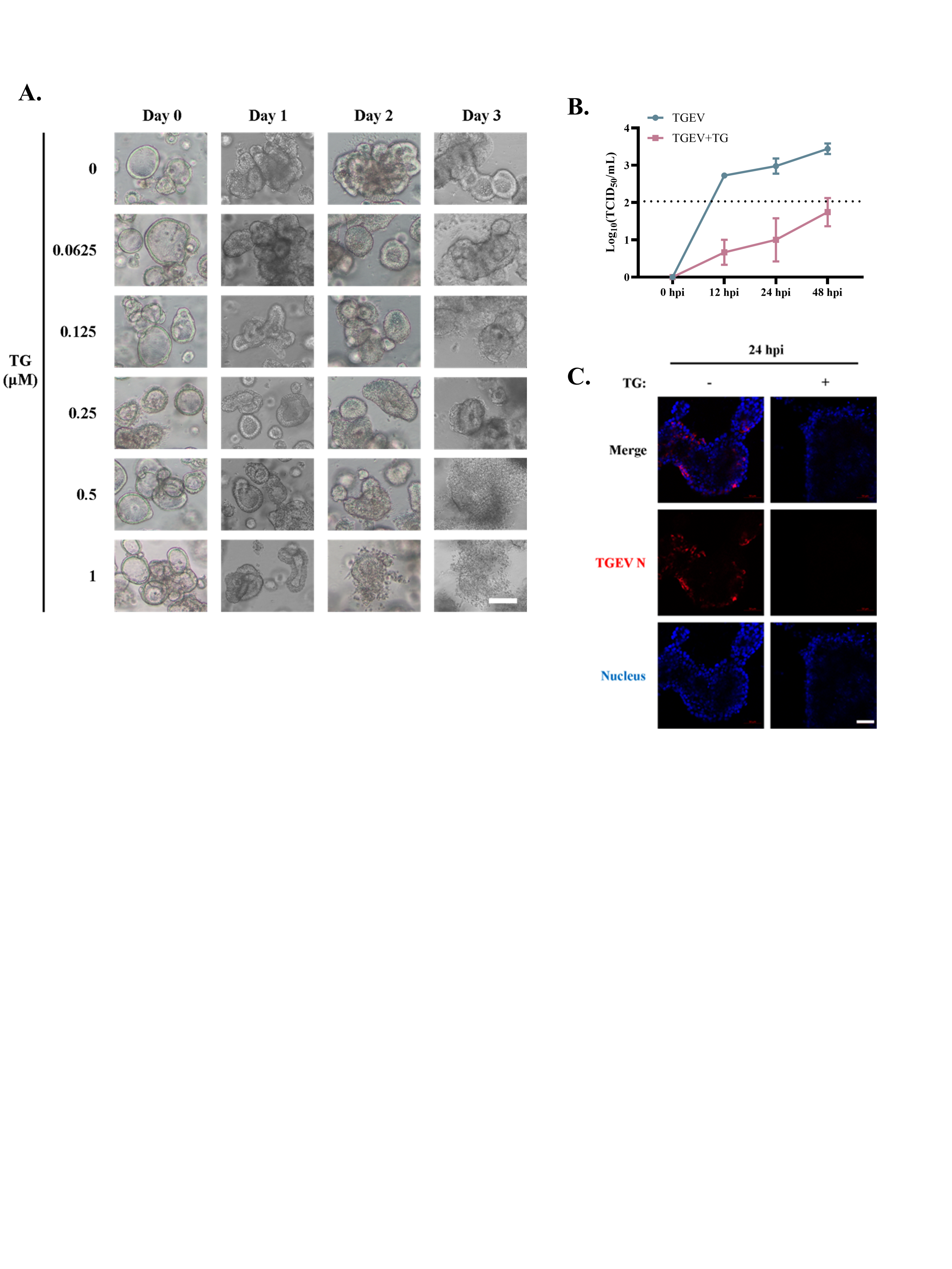

Supplement: Supplementary file 4 — Additional file 4. TG inhibits TGEV replication in 3D organoids. (A) 3D organoids were treated with TG at the indicated concentrations for three days. Organoids were imaged using bright-field microscopy (scale bar = 50 μm). (B) The supernatant of 3D organoids was harvested at 0, 12, 24, and 48 hpi (MOI = 5) to titrate TGEV using a TCID50 assay. The detection limit is shown as a dotted line. (C) IFA was performed to determine the impact of 0.25 μM TG on the reduction in TGEV-infected cells in 3D organoids (scale bar = 50 μm). All experiments were performed in triplicate. [file 13567_2024_1359_MOESM4_ESM.tif]

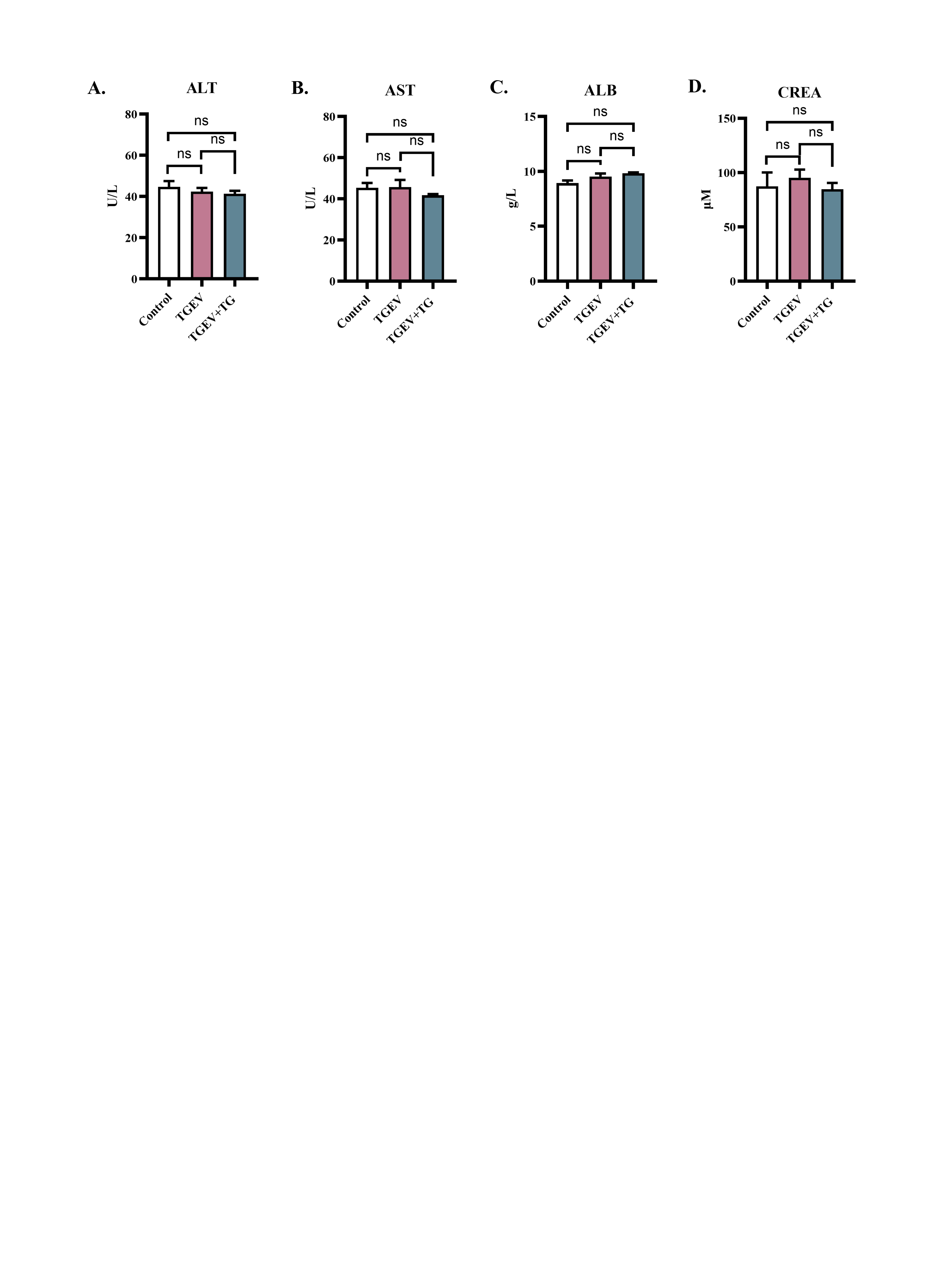

Supplement: Supplementary file 5 — Additional file 5. Short-term oral administration of TG does not cause liver or kidney toxicity in piglets. Neonatal piglets (n = 3) were orally challenged with TGEV at a dose of 3 × 10^7^ TCID50 per piglet. They were then orally administered TG at a dosage of 2 μg/kg at 1 and 12 hpi. Serum samples were collected at 24 hpi after the animals were sacrificed. The levels of serum CREA (A), AST (B), ALT (C), and ALB (D) were detected to evaluate TG-induced liver and kidney toxicity in the piglets. [file 13567_2024_1359_MOESM5_ESM.tif]

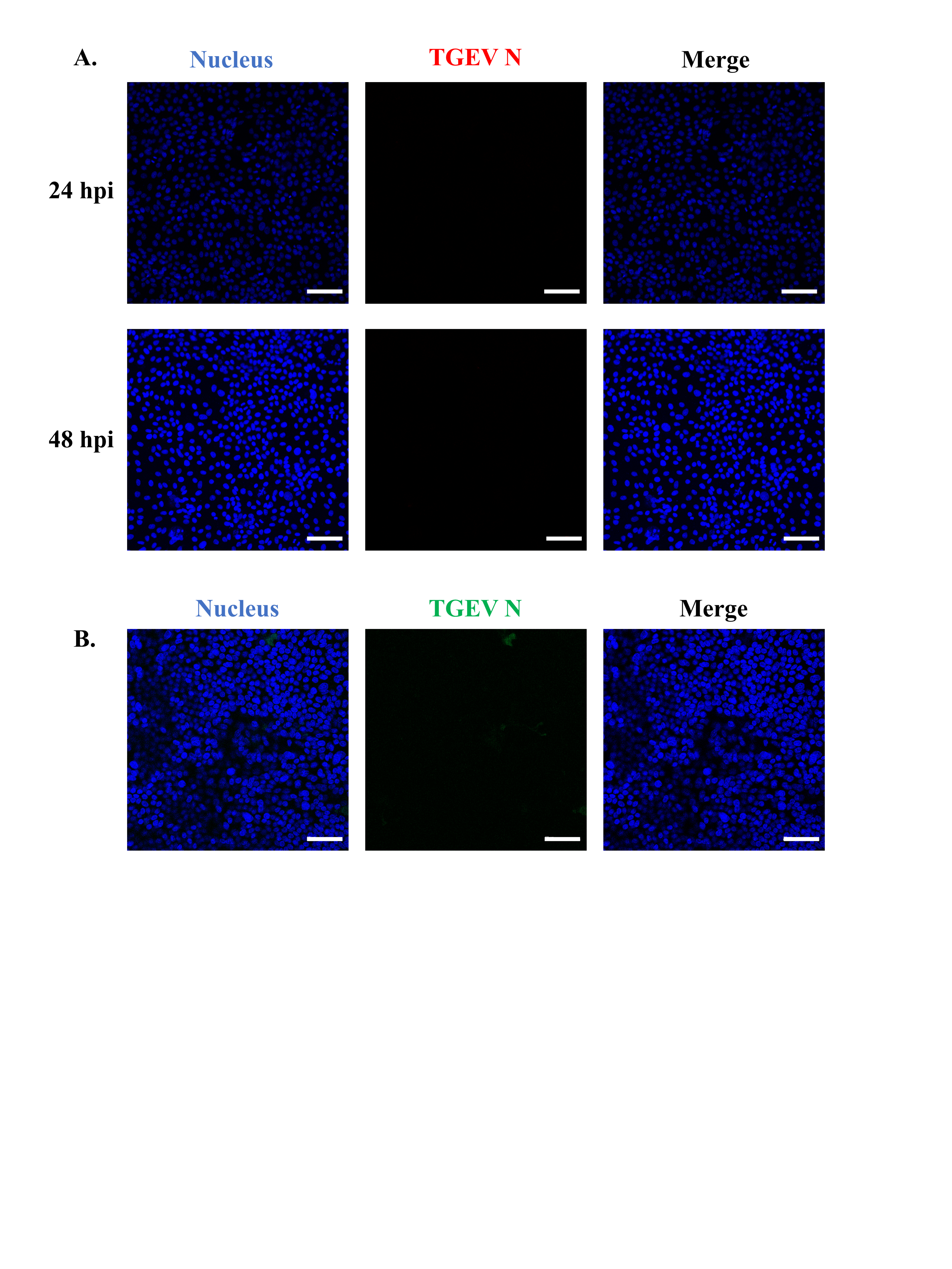

Supplement: Supplementary file 6 — Additional file 6. Immunostaining of TGEV N protein in the mock-infected group of IPEC-J2 cells and 2D intestinal organoid monolayers. (A) IFA was performed to detect TGEV in IPEC-J2 cells (scale bar = 50 μm). (B) IFA was performed to detect TGEV-infected cells on 2D intestinal organoid monolayers (scale bar = 50 μm). All experiments were performed in triplicate. [file 13567_2024_1359_MOESM6_ESM.tif]
